# A novel de novo *DDX3X* missense variant in a female with brachycephaly and intellectual disability: a case report

**DOI:** 10.1186/s13052-021-01033-4

**Published:** 2021-03-31

**Authors:** Giada Moresco, Jole Costanza, Carlo Santaniello, Ornella Rondinone, Federico Grilli, Elisabetta Prada, Simona Orcesi, Ilaria Coro, Anna Pichiecchio, Paola Marchisio, Monica Miozzo, Laura Fontana, Donatella Milani

**Affiliations:** 1 Research Laboratories Coordination Unit, Fondazione IRCCS Ca′ Granda Ospedale Maggiore Policlinico, Milan, Italy; 2 Fondazione IRCCS Ca′ Granda, Ospedale Maggiore Policlinico, Milan, Italy; 3 Child Neurology and Psychiatry Unit, IRCCS Mondino Foundation, Pavia, Italy; 4 Department of Brain and Behavioral Sciences, Università degli Studi di Pavia, Pavia, Italy; 5 Neuroradiology Department, IRCCS Mondino Foundation, Pavia, Italy; 6 Department of Pathophysiology and Transplantation, Università degli Studi di Milano, Milan, Italy; 7 Department of Health Science, Università degli Studi di Milano, Milan, Italy

**Keywords:** *DDX3X*, Rare disease, Intellectual disability, Polymicrogyria, Brachycephaly, Macroglossia, Case report

## Abstract

**Background:**

De novo pathogenic variants in the *DDX3X* gene are reported to account for 1–3% of unexplained intellectual disability (ID) in females, leading to the rare disease known as *DDX3X* syndrome (MRXSSB, OMIM #300958). Besides ID, these patients manifest a variable clinical presentation, which includes neurological and behavioral defects, and abnormal brain MRIs.

**Case presentation:**

We report a 10-year-old girl affected by delayed psychomotor development, delayed myelination, and polymicrogyria (PMG). We identified a novel de novo missense mutation in the *DDX3X* gene (c.625C > G) by whole exome sequencing (WES). The *DDX3X* gene encodes a DEAD-box ATP-dependent RNA-helicase broadly implicated in gene expression through regulation of mRNA metabolism. The identified mutation is located just upstream the helicase domain and is suggested to impair the protein activity, thus resulting in the altered translation of DDX3X-dependent mRNAs. The proband, presenting with the typical PMG phenotype related to the syndrome, does not show other clinical signs frequently reported in presence of missense *DDX3X* mutations that are associated with a most severe clinical presentation. In addition, she has brachycephaly, never described in female *DDX3X* patients, and macroglossia, that has never been associated with the syndrome.

**Conclusions:**

This case expands the knowledge of *DDX3X* pathogenic variants and the associated *DDX3X* syndrome phenotypic spectrum.

**Supplementary Information:**

The online version contains supplementary material available at 10.1186/s13052-021-01033-4.

## Background

About 1–3% of females with unexplained intellectual disability (ID) carry de novo pathogenic variants in the X-linked *DDX3X* gene (Xp11.4) [1]. Up to about 300 cases have been identified [2], both females with X-linked dominant inheritance, and very few males showing an X-linked recessive pattern of inheritance [1].

Patients with the rare *DDX3X* syndrome (MRXSSB, OMIM #300958) show a variable clinical presentation with different degrees of ID and/or developmental delay, neurological and behavioral defects, including microcephaly, hypotonia, epilepsy, movement disorders, autism spectrum disorder and aggressiveness. Brain malformations are also reported, and include corpus callosum hypoplasia, ventricular enlargement, and polymicrogyria (PMG). Additional clinical features, among which facial dysmorphisms and sensory deficits, may also be present.

To date, all reported pathogenic *DDX3X* variants identified in affected females are de novo loss of function, leading to haploinsufficiency and, probably, to embryonic lethality in males. Most of *DDX3X* mutations reported in males are maternally inherited missense variants. In these families, males showed borderline to severe ID and carrier females were unaffected and expressed a DDX3X protein retaining a partial functionality [1]. Several hypotheses have been evaluated to explain the gender-specific pathogenicity, including a skewing X-inactivation pattern, but no definitive conclusions have been drawn. Kellaris et al. [3] proposed that hypomorphic *DDX3X* variants may be viable in hemizygous males and do not cause clinical phenotype in female carriers. However, few de novo *DDX3X* mutations have been identified in males [4]. Although these male patients share many of the clinical features with affected females, some distinct clinical phenotypes can depend on the gender of the patient and on the pathogenicity of the variant. These findings suggest that the *DDX3X* gene exerts dose-dependent and gender-specific effects in normal development and disease [5]. Indeed, Snijders Blok et al. [1] reported a family with recurrent miscarriages of unknown gender and a male viable pregnancy, terminated after the identification of severe congenital anomalies by ultrasound imaging. By whole exome sequencing (WES) a missense mutation in the *DDX3X* gene was detected in the fetus, suggesting a potential germline mosaicism in the mother.

The *DDX3X* gene encodes a DEAD-box ATP-dependent RNA-helicase broadly implicated in gene expression through regulation of mRNA metabolism [6]. It acts as a translational regulator of target mRNAs with highly structured 5′ untranslated regions (UTRs) and it is involved in repeat-associated non-AUG translation [7]. DDX3X also takes part in stress response and stress granule assembly, innate immune signaling, mitotic chromosome segregation and it can also exert a role in tumorigenesis [8]. In addition, it is thought to be an essential factor in the RNA-interference pathway [9], and it is a key regulator of the Wnt/β-catenin pathway [10].

Despite the ubiquitous expression of the DDX3X protein, high expression levels are detected in all the cortical layers of the embryonic brain, consistent with its crucial role in cortical development during neurogenesis [10] and, as a consequence, *DDX3X* mutations are reported to impact on neuronal function [4]. In particular, defective neurite outgrowth and neural progenitor differentiation/migration could account for brain malformations and the consequent characteristic clinical phenotypes of the *DDX3X* syndrome [5].

Here, we report a 10-year-old girl with delayed psychomotor development, delayed myelination, bilateral frontal PMG and thin body and splenium of the corpus callosum. WES allowed the identification of the novel de novo *DDX3X* mutation c.625C > G (p.His209Asp), leading to a diagnosis of *DDX3X* syndrome and expanding the number of *DDX3X* pathogenic variants and their associated phenotypic spectrum.

## Case presentation

The proband was referred to our laboratory by the pediatric geneticists of our Institution, Fondazione IRCCS Ca′ Granda Ospedale Policlinico (Milan). Appropriate written informed consent was obtained from all family members.

**Fig. 1:**
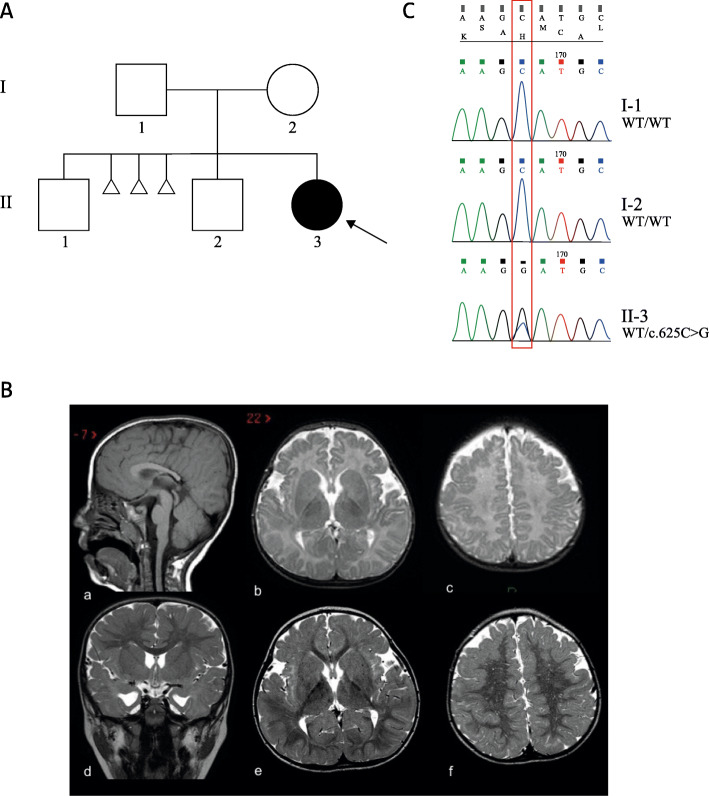
**A**) Pedigree of the family. **B**) Brain MRI with sagittal T1-weighted sequence (a) and axial (b-c-e-f) and coronal (d) T2-weighted sequences, performed respectively at 6 months of age (b-c) and 2 years old (a-d-e-f). A thin posterior corpus callosum is evident (a), involving the body, the isthmus and the splenium, with consequent dysmorphic temporal horns (d). A delayed myelination is evident at 6 months (b-c) as lack of diminution of signal intensity within the centrum semiovale, bilaterally (c) and in the corpus callosum (b); in the control MRI, myelination is complete (e-f), with evidence of numerous perivascular spaces within the white matter (f). Frontal cortical thickening is evident, with irregularity of the cortical-white matter junction (c-f) in both hemispheres, consistent with PMG . **C**) Sequencing chromatograms of DNA samples from the proband and the parents. The position of the novel *DDX3X* variant identified is indicated by a red box. The proband is confirmed to be heterozygous for the *DDX3X* c.625C > G variant, while parents are wild-type

Patient II-3 (Fig. 1A), who is currently 10 years old, is the third-born of healthy parents with a doubtful distant consanguinity (beyond the second generation). The two older siblings (II-1 and II-2, of 16 and 14 years old respectively) are both healthy. Between the first and second pregnancy three miscarriages of unknown gender occurred, but no additional information is available. The family history is negative for genetic conditions.

The patient was born at 38 + 5 gestational weeks, after a normal pregnancy, characterized by regular growth, morphology and fetal movements. She was born by cesarean delivery scheduled for previous cesarean section.

Clinical signs are reported in Table 1. At birth, the child was 2740 g (25th centile), 49 cm long (50th centile), presented an occipitofrontal circumference of 34 cm (50th centile) and an Apgar score of 10–10. Absence of skin adnexa (eyelashes, eyebrows and nails) and difficult scarring of the umbilical stump are referred. In addition, a bilateral II-III toe syndactyly and a small left preauricular tag were evident. Ultrasounds revealed normal abdominal and cerebral morphology.

**Table 1:** Clinical features of II-3 compared to the characteristic clinical sings reported for the *DDX3X* syndrome and relative prevalence

***DDX3X*** syndrome clinical signs (relative prevalence)	Clinical signs of II-3
**Development**	
Developmental delay (106/106) [10]	+
Intellectual disability (106/106) [10]	+
Language delay (38/75) [10]	+
**Growth**	
Failure to thrive (13/44) [11]	+
Short stature (1/6) [11]	–
Microcephaly (39/107) [11]	–
Brachycephaly (3/11) [11]	+
**Neurologic/behavioral**	
Seizures (24/116) [10]	EEG anomalies
Hypotonia (66/116) [10]	+
Hypertonia/spasticity (9/78) [11]	–
Mixed hypo and hypertonia (31/93) [10]	–
Sleep disturbance (2/6) [11]	+
Movement disorders/spasticity in the legs (22/49) [11]	–
Behavior disorders/autism spectrum disorder/aggression (24/49) [11]	–
Hyperreflexia (9/78) [11]	+
**Brain MRI**	
Polymicrogyria (11/89) [10]	+ (anterior)
Corpus callosum hypoplasia/agenesis (76/105) [11]	+
Ventricular enlargement (27/105) [11]	–
Key-hole shaped temporal horns (32/89) [10]	–
Colpocephaly (3/89) [10]	–
Delayed myelination/decreased cortical white matter (50/89) [10]	+
Small pons (11/89) [10]	–
Small inferior vermis (6/89) [10]	–
**Sensory**	
Vision problems (strabismus, coloboma, astigmatism, nystagmus) (29/92) [10]	+
Hearing problems (11/114) [11]	–
**Facial dysmorphisms**	
Short/down-slanting palpebral fissure length (2/6) [11]	–
Hypertelorism/telecanthus (6/36) [11]	+
Epicanthal folds (1/6) [11]	–
Elongated/flattened/triangular face (9/36) [11]	+
High/broad forehead (8/36) [11]	+
Wide nasal bridge/bulbous tip (9/36) [11]	+
Short/narrow nose, anteverted nares (11/36) [11]	–
Micrognathia (2/6) [11]	+
High arched palate (4/6) [11]	ND
Thin upper lip (4/6) [11]	+
Low set/protruding/wide ears (2/6) [11]	+
Smooth/long philtrum (3/6) [11]	–
Cleft lip/palate (3/44) [11]	–
Macroglossia (ND)	+
**Other**	
Congenital cardiac defects (13/90) [10]	–
Precocious puberty (11/94) [10]	–
Feeding difficulties (gastro-esophageal reflux/swallowing) (3/6) [11]	+
Joint hyperlaxity (14/44) [11]	–
Scoliosis (15/94) [10]	–
Malformations of the hands (1/6) [11]	+
Skin pigmentation anomalies (16/44) [11]	–
Loss/reduced subcutaneous fat (2/6) [11]	+

*Abbreviations: ND* Not defined

In the first months of life, a severe gastro-esophageal reflux with initial weight loss and sleep disturbance was reported, which improved after the introduction of proton-pump inhibitors therapy.

Growth was regular, but psychomotor development was severely delayed, with independent walking and babbling acquired towards 6 years of age.

Evaluation at 6 years old revealed the following cranio-facial dysmorphisms: brachycephaly and a flattened-triangular-asymmetrical face characterized by micrognathia, mild hypertelorism, wide and prominent nose, short philtrum, thin lips and macroglossia. She also presented a slight facial grimacing, large and anteverted ears and small left ear tag. Growth parameters were normal, with a weight of 16.5 kg (10-25th centiles), a height of 111 cm (50-75th centile), head circumference of 50.5 cm (25-50th centile).

Brain MRI performed at 6 months of age showed delayed myelination (improved at the second MRI performed at 2 years old), bilateral frontal PMG and thin body and splenium of the corpus callosum (Fig. 1B). Electroencephalogram (EEG) showed sharp-wave anomalies on the rear right regions. She also presents hyperopia and divergent strabismus.

Karyotype, aCGH and mutational analysis of the *ADGRG1* gene (associated to bilateral frontoparietal PMG resulted normal. An NGS panel for Rett syndrome (including the *MECP2, CDKL5, FOXG1, MEF2C, SCN1A, UBE3A, PCDH19, STXBP1* genes) was also performed, and no pathogenic variants were identified.

Since no other clinical suspicions were hypothesized, the proband and her parents underwent WES on DNA extracted from peripheral blood leukocytes. Trio WES and variants interpretation according to the pedigree and the clinical features allowed the identification of the novel missense variant c.625C > G (p.His209Asp) in exon 7 of the *DDX3X* gene. No other candidate variants were found, and the identified variant was confirmed by Sanger sequencing. The absence of the mutation in the healthy mother confirmed the hypothesis of a de novo inheritance (Fig. 1C). However, considering the occurrence of three miscarriages between the first and the second pregnancy, we cannot exclude the possibility that also the aborted fetuses could have carried the c.625C > G mutation as a consequence of a possible germline mosaicism in the mother.

The identified *DDX3X* variant is neither reported in ClinVar nor in HGMD Professional 2020.1. To determine the impact of this amino acid substitution, the pathogenic score was evaluated by thirteen different *in-silico* predictors, and it was predicted to be damaging by nine of them (Supplementary Table 1). This score is consistent with the amino acid substitution from a positively charged histidine to a negatively charged aspartic acid, in a highly conserved residue.

## Discussion and conclusions

WES analysis in a 10-year-old girl with delayed psychomotor development, characteristic facial dysmorphisms and brain malformations, including delayed myelination, bilateral frontal PMG and thin body and splenium of the corpus callosum, allowed the identification of the novel missense variant c.625C > G (p.His209Asp) in exon 7 of the *DDX3X* gene, suggesting a diagnosis of *DDX3X* syndrome. The absence of the mutation in the healthy mother confirmed the hypothesis of a de novo inheritance (Fig. 1C), as reported for all *DDX3X* mutations found in females [1]. However, considering the occurrence of three miscarriages between the first and the second pregnancy, we cannot exclude the possibility that also the aborted fetuses could have carried the c.625C > G mutation as a consequence of a possible germline mosaicism in the mother. This is consistent with the association of *DDX3X* mutations to miscarriage recurrence and aborted male fetuses [1]. However, the unavailability of information on the aborted fetuses and the impossibility to assess the presence of the germline mosaicism in the mother prevents us to confirm this hypothesis.

**Fig. 2:**
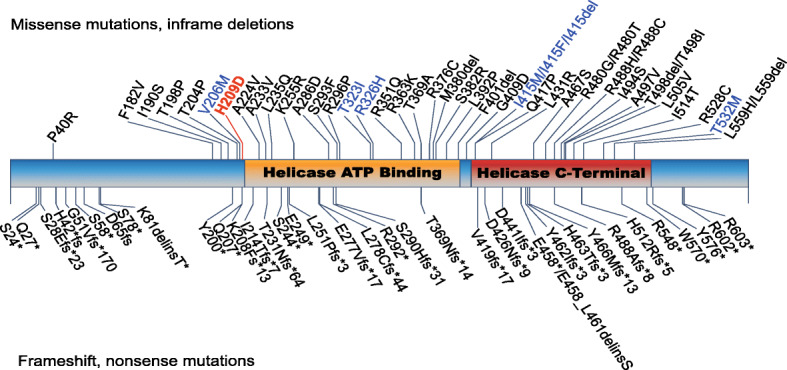
Schematic representation of the DDX3X protein domains and mapping of mutations already reported in patients affected by the *DDX3X* syndrome (adapted from [10]). Missense mutations and in-frame deletions are reported on top, while frameshift and nonsense mutations are annotated on the bottom. Mutations reported in patients with PMG are displayed in blue. The His209Asp variant identified in II-3 is reported in red

The novel c.625C > G variant involves the amino acidic position 209, which maps 2 bp upstream the helicase ATP binding domain (Fig. 2). Most reported *DDX3X* mutations map within the two helicase domains impairing the protein helicase activity, with a dominant negative mechanism [10]. The novel His209Asp variant is close to the Val206Met, already reported in a patient with the *DDX3X* syndrome [10]. Despite no functional tests have been performed to confirm the pathogenic effect of this novel mutation, according to in silico predictions and literature data on the close Val206Met and other missense mutations that fall within or in proximity of the DDX3X helicase domain [10], we hypothesize that also the His209Asp variant leads to an impaired helicase activity. Deficient DDX3X helicase activity results in the enzyme inability to release the bound RNA after ATP hydrolysis and the altered translation of DDX3X-dependent targets, in particular mRNAs containing highly structured 5′ UTRs and/or high GC content [12]. This leads to the sequestration of RNAs and RNA binding proteins, and the formation of aberrant ribonucleoprotein (RNP) granules containing newly synthetized proteins and/or stalled polysomes [10]. The defective translation of DDX3X-target transcripts has been proposed to lead to impaired neurogenesis, likely affecting embryonic cortical development [10]. This mechanism may also account for the more severe phenotypes associated with missense mutations, compared to nonsense and frameshift (presumed loss of function) lesions, that affect translation of some targets but do not induce granule formation, resulting in a less severe clinical presentation [10]. This pathomechanism may also apply to the His209Asp mutation here reported, as suggested, not only by the affected protein domain, but also by the PMG phenotype of the proband herein presented.

According to the recent studies on genotype-phenotype correlations in the *DDX3X* syndrome and underlying pathogenetic mechanisms [10], patients with severe cerebral defects, including complete or partial absence of the corpus callosum and PMG (frequently associated to microcephaly), presented missense mutations or single amino acid deletion hotspot mutations, including the nearby Val206Met (Fig. 2). These mutations are also associated to more complex clinical presentations, including epilepsy, autism spectrum disorder, severe intellectual disability, and cardiovascular anomalies, not present in patients without PMG [10]. This evidence thus suggests that these variants are likely to lead to a more severe dominant negative phenotype, while loss of function mutations are associated to a milder spectrum of clinical traits with no signs of PMG. Despite the proband herein described presents with PMG, she does not show other clinical traits reported in most severe cases of *DDX3X* syndrome, such as microcephaly, congenital cardiac defects and epilepsy (she only presents EEG anomalies) (Table 1). In addition, she shows reduced subcutaneous fat, already reported in two other patients *DDX3X* syndrome patients [11], and she is the first female patient presenting with brachycephaly (which has so far been described only in few males [1]) (Table 1). She also has macroglossia, an additional clinical sign that has never been associated with the *DDX3X* syndrome before (Table 1).

In conclusion, these distinctive clinical features, together with the presence of PMG, but no other signs associated to a severe phenotype, furtherly expand genotype-phenotype correlations of *DDX3X* missense mutations.

This case report emphasizes the clinical utility of WES in ending the diagnostic odyssey of individuals with unexplained ID.

## Supplementary Information

**Additional file 1: Supplementary Table 1.** Prediction of pathogenicity of the identified *DDX3X* variant by *in silico* tools.

## Data Availability

The datasets used and/or analyzed during the current study are available from the corresponding author on reasonable request.

## Abbreviations

DDX3X: DEAD-Box Helicase 3 X-Linked
ID: Intellectual disability
MRXSSB: Mental retardation, X-linked 102
PMG: Polymicrogyria
WES: Whole exome sequencing
MRI: Magnetic resonance imaging
EEG: Electroencephalogram
aCGH: Array-Comparative Genomic Hybridization
