# Supplementary material for: A novel de novo DDX3X missense variant in a female with brachycephaly and intellectual disability: a case report
Source: Ital J Pediatr. 2021 Mar 31;47:81. doi: 10.1186/s13052-021-01033-4 (PMC8011215; doi:10.1186/s13052-021-01033-4)
Supplement: Supplementary file 1 — Additional file 1: Supplementary Table 1. Prediction of pathogenicity of the identified DDX3X variant by in silico tools. [file 13052_2021_1033_MOESM1_ESM.docx]

**Supplementary material**

Supplementary Table 1. Prediction of pathogenicity of the identified *DDX3X* variant by *in silico* tools.

| **Predictor** | **Damaging score range** | **Score and functional impact** |
| --- | --- | --- |
| SIFT | from 0 to 0.05 | 0.008 (damaging) |
| Polyphen-2 HVAR | from 0.447 to 1 | 0.859 (probably damaging) |
| LRT | from 0 to 0.0005 | 0 (damaging) |
| MutationTaster | from 0.5 to 1 | 1 (disease-causing) |
| MutationAssessor | > 0.935 | 0.695 (neutral) |
| FATHMM | < -1.5 | 2.53 (tolerated) |
| PROVEAN | from 14 to -2.5 | -5.79 (damaging) |
| VEST3 | from 0.5 to 1 | 0.699 (damaging) |
| CADD | from 20 to 39 | 26.9 (damaging) |
| DANN | from 0.96 to 1 | 0.993 (damaging) |
| fathmm_MKL | from 0.5 to 1 | 0.91 (damaging) |
| MetaSVM | from 0.5 to 1 | -0.905 (tolerated) |
| MetaLR | from 0.5 to 1 | 0.140 (tolerated) |
